# Self-sustained activity in neural networks: influence of network topology and cell types

**DOI:** 10.1186/1471-2202-14-S1-P411

**Published:** 2013-07-08

**Authors:** Diogo PC Vieira, Rodrigo FO Pena, Antonio C Roque

**Affiliations:** 1 Departamento de Física, FFCLRP, Universidade de São Paulo, Ribeirão Preto, SP, 14040-901, Brazil

## 

The cerebral cortex exhibits spontaneous and structured collective activity patterns even in the absence of external stimuli [1]. A possible question that can be made given this evidence is: how does the topology of the cortical network together with the individual properties of the neuronal types that populate it affect this self-sustained neural activity? Anatomical evidence suggests that cortical architecture has modular and hierarchical design [2]. Based on their response patterns to intracellular current injection, cortical neurons may be classified into 5 main electrophysiological classes [3]: regular spiking (RS), intrinsically bursting (IB), chattering (CH), fast spiking (FS) and with low threshold spikes (LTS). Cells from the first 3 types are excitatory and cells from latter 2 are inhibitory [3]. In this work we used a hierarchical and modular network model composed of excitatory and inhibitory neurons to study the joint effect of (i) modularity level and (ii) combination of excitatory and inhibitory cell types on self-sustained network activity.

Our hierarchical and modular network was constructed using a top-down method [4] starting with a random network of 1024 cells with connection probability of 0.01. The ratio of excitatory to inhibitory neurons was 4:1. Neurons were modeled using Izhikevich's neuron model [5] with parameters adjusted to reproduce the firing behaviors of the 5 cell types. Our model has 2 synaptic conductances (*g*_e_, *g_i_*), representing excitatory and inhibitory synapses. After a pre-synaptic event, these synaptic conductances are increased by constants Δ*g_e_*, Δ*g_i_*. Otherwise, they decay according to first-order linear kinetics with characteristic times *τ_e _*= 5 ms and *τ_i _*= 6 ms. Using the modularization method of [4] (with rewiring probabilities *R_i _*= 1 and *R_e _*= 0.9), we generated networks with 4 modularity levels (0-3). For each level we generated 6 networks given by the possible combinations of the 3 excitatory neuron types with the 2 inhibitory types.

Our experimental protocol consisted in stimulating a network by applying white noise to all neurons for 200 ms. After the noise was switched off the simulation was allowed to run for an extra 4800 ms. We performed this experiment for 50 realizations of each network (to calculate averages) and for different combinations of the parameters (Δ*g*_e_, Δ*g*_i_). From the resulting data we constructed, for each network configuration, a Δ*g*_e_-Δ*g*_i _diagram plotting the time of last network spike. We defined a threshold for this time (~4500 ms) beyond which we assumed the network as having self-sustained activity. For each diagram, we measured the fraction of its total area for which self-sustained activity existed according to our criterion. This fraction, called FTA, was our measure of self-sustained network activity.

Our results show that (1) FTA increases with the modularity level; (2) networks with RS excitatory cells have the highest FTA variability with modularity level; and (3) networks with CH excitatory neurons have the smallest FTA variability with modularity. For these latter networks, FTA reached maximum value for level-1 modularity. Our results show a strong dependency of self-sustained activity on both the modularity level and types of excitatory and inhibitory cells. They suggest that modular architecture favors self-sustained activity and that networks with most of excitatory cells of the RS class exhibit the widest range of self-sustained regimes.
